# Development and validation of a nomogram for pressure injury risk prediction in stroke patients: a retrospective cohort study

**DOI:** 10.3389/fneur.2025.1593707

**Published:** 2025-09-09

**Authors:** Tang Haiyan, Zhong Quanzhen, Liao Tingting, Fu Qing, Xie Yulei, Lv Zewei, Zhou Mijuan, Huang Bo

**Affiliations:** ^1^Rehabilitation Department of Zigong First People’s Hospital, Zigong, Sichuan, China; ^2^Rehabilitation Department, Affiliated Hospital of North Sichuan Medical College, Nanchong, Sichuan, China; ^3^Otolaryngology, Zigong First People's Hospital, Zigong, Sichuan, China

**Keywords:** stroke, pressure ulcer, nomogram, risk prediction, retrospective study

## Abstract

**Study design:**

Retrospective cohort study.

**Objective:**

This study aimed to identify independent risk factors for pressure injury (PI) during the post-stroke recovery phase, develop and validate a nomogram prediction model to facilitate the identification of high-risk individuals for PI, and establish a theoretical framework for optimizing clinical intervention strategies.

**Methods:**

Retrospective clinical data were collected from 284 hospitalized stroke patients in the recovery phase (including 85 PI cases) at the Affiliated Hospital of North Sichuan Medical College between January 2018 and December 2022. Participants were randomly allocated into training (70%) and internal validation (30%) cohorts. An external validation cohort comprising 60 stroke patients (30 PI cases) from Zigong First People’s Hospital (January 2023–January 2024) was additionally analyzed. Univariate analysis and LASSO regression were utilized to screen independent PU risk factors, followed by nomogram construction. Model performance was evaluated using the C-index, calibration curves, and Decision Curve Analysis (DCA). Comparative analyses were conducted against the Braden scale (Model 2) and a combined model incorporating the Braden scale (Model 3).

**Results:**

Independent risk factors for PI in post-stroke recovery patients included hemorrhagic stroke subtype, advanced age, hypoalbuminemia, elevated leukocyte counts, and low Activities of Daily Living (ADL) scores. The nomogram model incorporating these five predictors demonstrated AUC values of 0.902 (training cohort), 0.935 (internal validation), and 0.936 (external validation), exceeding the predictive capacity of individual variables: stroke type (AUC = 0.642), age (AUC = 0.756), albumin level (AUC = 0.754), leukocyte count (AUC = 0.712), and ADL score (AUC = 0.839). Calibration curves indicated strong concordance between predicted and observed outcomes, while DCA confirmed substantial clinical net benefit. The Braden scale (AUC = 0.817) exhibited inferior predictive performance compared to our model, and the combined model (AUC = 0.901) showed no significant improvement, underscoring the parsimony and clinical utility of the proposed nomogram.

**Conclusion:**

The nomogram developed in this study for predicting PIs in stroke recovery patients demonstrates high accuracy and discrimination, facilitating the early identification of high-risk individuals and aiding in the formulation of personalized intervention strategies.

## 1 Background

Stroke is a leading cause of death and disability worldwide, and the extent of functional recovery significantly impacts the quality of life for survivors (1). Pressure injury (PI) develop as a result of prolonged, unrelieved pressure, leading to localized damage to the skin, mucous membranes, and/or underlying tissues (2). Stroke survivors face an increased risk of PI due to multiple factors, including limited mobility, motor and sensory impairments, and compromised tissue perfusion (3). This risk is particularly pronounced during the recovery phase, when patients transition from hospital care to community rehabilitation, often leading to higher rates of PI occurrence (4).

In hospital settings, the prevalence of PIs ranges from 1 to 3%, whereas in community or home environments, this figure can rise to between 3.3 and 11.1% (5). The development of PI not only diminishes patients’ quality of life and increases economic burdens but also significantly elevates morbidity and mortality rates (6, 7). Various tools, such as the Braden scale, Activities of Daily Living (ADL) assessments, adipose tissue thickness measurements, and imaging techniques, are commonly used to predict the risk of PI. However, their effectiveness is often limited due to factors such as subjectivity, data gaps, and variability in sensitivity to lighting conditions (8, 9).

Current PI prediction methods tend to be complex and broad, aiming to encompass diverse populations without addressing specific conditions adequately (5, 10). In stroke recovery patients, the risk factors for PI development may differ from those in other groups, influenced by unique characteristics associated with stroke, such as type, age, body mass index (BMI), ADL scores, and muscle tone status (3, 11). This study aims to develop and validate an integrated predictive model by synthesizing multidimensional demographic characteristics, clinical treatment parameters, and laboratory test indicators, with the objective of accurately identifying risk predictors for PI development among stroke rehabilitation patients during hospitalization. Through the establishment of a risk stratification assessment system, healthcare professionals can formulate personalized intervention strategies based on evidence-based medical principles, thereby effectively preventing or reducing the incidence of PI.

## 2 Materials and methods

### 2.1 Participants data collection

**Figure 1:**
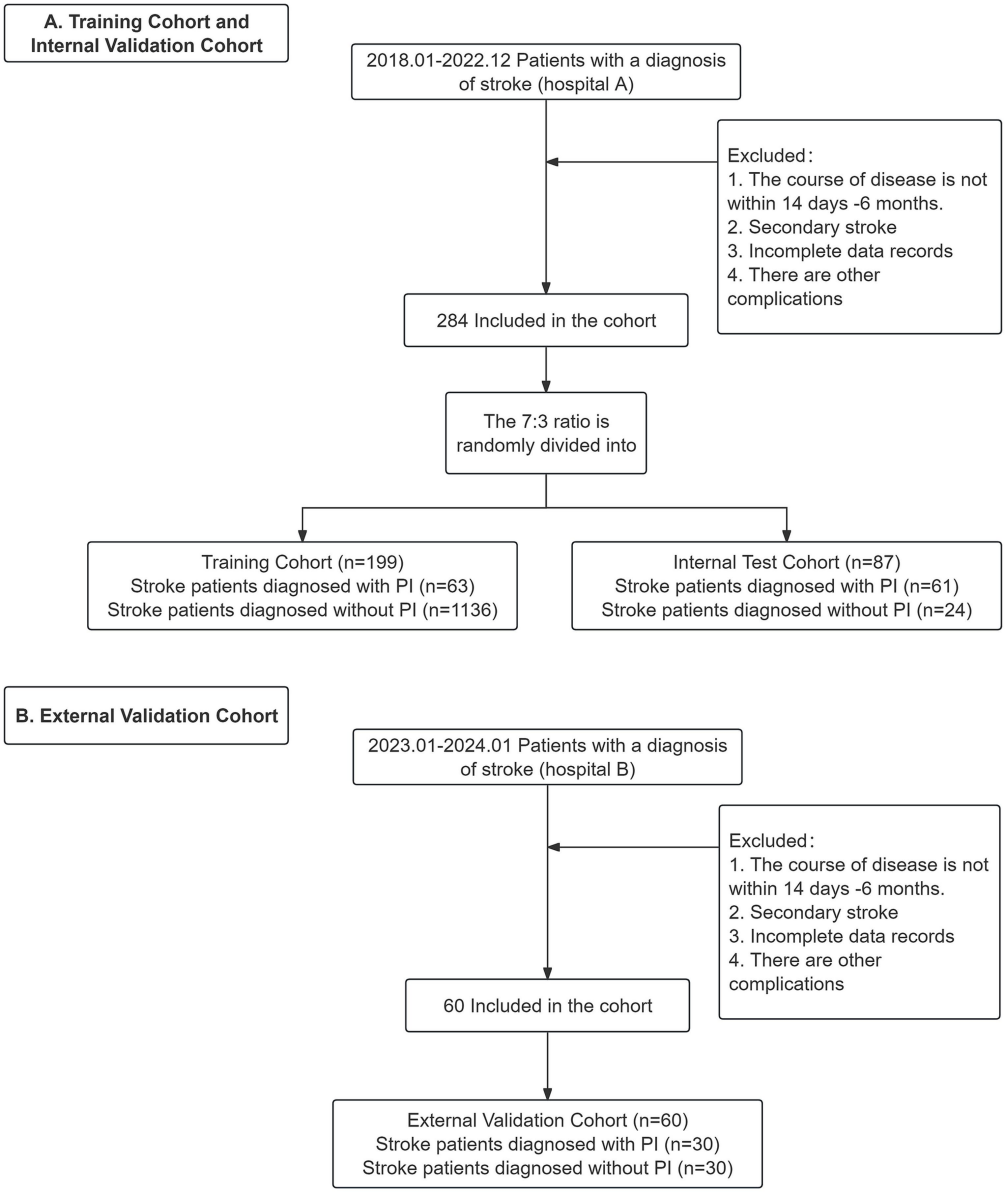
Training and validation cohorts used to develop the prediction models for pressure injury (PI) after stroke. A. Training Cohort andInternal Validation Cohort. B. External Validation Cohort.

The flowchart of the study process is shown in Figure 1. In this retrospective study, all datasets were derived from the electronic medical records of two hospitals in China. Specifically, data from 87 stroke patients in the recovery phase, diagnosed with PI between January 2018 and December 2022 at the Affiliated Hospital of North Sichuan Medical College, along with data from 197 stroke patients without PI, collectively formed the training and internal validation cohort. Additionally, data collected between January 2023 and January 2024 from 60 stroke patients (including 30 patients with PI) at Zigong First People’s Hospital, served as the external validation cohort. Ethical approval was obtained from the Institutional Review Boards of North Sichuan Medical College (No. 2022ER363-1) and Zigong First People’s Hospital (No. M-022) and participants were informed and consent was obtained prior to the commencement of the study. The research adhered strictly to the principles outlined in the Declaration of Helsinki.

Inclusion Criteria:

(1) All participants were adult patients with a first – time stroke (inclusive of ischemic and hemorrhagic subtypes) confirmed by imaging studies, in the recovery phase (14–6 months post – onset duration) (12, 13).
(2) Participants with PIs met the international NPUAP/EPUAP diagnostic criteria for Stage II or higher (14).
(3) Participants were aged ≥ 18 years.
(4) Participants had complete baseline data, including demographic characteristics, laboratory indicators, and data on predictive factors.
(5) Participants with recurrent stroke were excluded.

Exclusion Criteria:

(1) Participants in the acute phase (<14 days post – onset) or the late – sequelae phase (>6 months post – onset) of stroke.
(2) Participants with a history of recurrent stroke (≥2 stroke events).
(3) Participants with incomplete records of key predictive variables (e.g., albumin levels, ADL scores).
(4) Participants with a history of PI unrelated to stroke or chronic illnesses affecting skin integrity (e.g., diabetic foot, venous ulcers).

### 2.2 Variables included in the study

We collected the following demographic and clinical data: presence of PIs, stroke subtype, age, sex, BMI, diabetes, hypertension, chronic obstructive pulmonary disease (COPD), disorders of consciousness (DOC), coronary artery disease (CAD), muscle tone, Activities of Daily Living (ADL) scores, albumin levels, white blood cell counts, and Braden scale scores.

### 2.3 Sample size calculation

The sample size for the predictive model was determined using the 5 events per variable (EPV) method, requiring at least 5 samples for each variable. In this study, 14 variables were included, necessitating a minimum of 70 samples (14 × 5). The training cohort data set met this requirement.

### 2.4 Statistical analysis

Multiple imputation was employed for data preprocessing. The dataset from the Affiliated Hospital of North Sichuan Medical College was randomly divided into a training cohort and an internal test cohort at a 7:3 ratio, while the data from Zigong First People’s Hospital served as the external validation cohort. Non-normally distributed data are presented as medians with interquartile ranges. For univariate analyses, categorical variables were assessed using chi-square tests or Fisher’s exact tests, while continuous variables were analyzed using t-tests or rank-sum tests. In the training cohort, multivariable analysis was conducted using LASSO logistic regression to identify independent risk factors and construct a predictive nomogram. The performance of the nomogram was assessed using receiver operating characteristic (ROC) curves and calibration plots, with the area under the curve (AUC) ranging from 0.5 (no discrimination) to 1 (perfect discrimination). Decision curve analysis (DCA) was also employed to determine the threshold for net benefit. A *p*-value of less than 0.05 was considered statistically significant. All statistical analyses were performed using R software (version 4.2.2) and MSTATA software^1^.

## 3 Results

### 3.1 Demographic characteristics

The study enrolled 344 stroke recovery patients with a mean age of 72 ± 13 years, comprising 40.9% females (*n* = 141) and 59.1% males (*n* = 203). Participants were stratified into training (*n* = 199), internal validation (*n* = 85), and external validation cohorts (*n* = 60) in a 7:3 ratio. No statistically significant differences were observed across cohorts in age (*p* = 0.168), gender distribution (*p* = 0.602), COPD (*p* = 0.575), DOC (*p* = 0.219), CAD (*p* = 0.508), white blood cell counts (*p* = 0.83), or Braden scale scores (*p* = 0.462).

The external validation cohort exhibited distinct clinical profiles: hemorrhagic stroke was more prevalent (63.3% vs. 15.6% in training cohort, *p* < 0.001) with lower rates of diabetes (18.3% vs. 42.7%) and hypertension (36.7% vs. 58.8%, both *p* < 0.001). Muscle tone abnormalities were significantly more frequent (25.0% vs. 4.5%, *p* < 0.001). Laboratory parameters showed comparable albumin levels (36 ± 7 vs. 37 ± 6 vs. 36 ± 7, *p* = 0.47) and ADL scores (48 ± 31 vs. 51 ± 31 vs. 54 ± 31, *p* = 0.391) across groups. These findings underscore substantial regional variability in pressure injury risk profiles among stroke recovery patients, emphasizing the necessity for multicenter validation in predictive model development (Table 1).

**Table 1:** Patient demographics and baseline characteristics.

Characteristic	Cohort			*p*-value^2^
	Training cohort, *N* = 199^1^	Internal validation cohort, *N* = 85^1^	External validation cohort, *N* = 60^1^	
Stroke subtype				<0.001
Cerebral hemorrhage	31 (15.6%)	12 (14.1%)	38 (63.3%)	
Cerebral infarction	168 (84.4%)	73 (85.9%)	22 (36.7%)	
Age (mean ± SD)	70 ± 14	73 ± 12	69 ± 17	0.168
Genders				0.602
Female	80 (40.2%)	33 (38.8%)	28 (46.7%)	
Male	119 (59.8%)	52 (61.2%)	32 (53.3%)	
BMI (mean ± SD)	23.0 ± 3.3	23.0 ± 3.4	22.2 ± 4.9	0.32
Diabetes				0.001
No	114 (57.3%)	47 (55.3%)	49 (81.7%)	
Yes	85 (42.7%)	38 (44.7%)	11 (18.3%)	
Hypertensive				0.001
No	82 (41.2%)	29 (34.1%)	38 (63.3%)	
Yes	117 (58.8%)	56 (65.9%)	22 (36.7%)	
COPD				0.575
No	190 (95.5%)	79 (92.9%)	56 (93.3%)	
Yes	9 (4.5%)	6 (7.1%)	4 (6.7%)	
DOC				0.219
No	154 (77.4%)	64 (75.3%)	52 (86.7%)	
Yes	45 (22.6%)	21 (24.7%)	8 (13.3%)	
CAD				0.508
No	166 (83.4%)	69 (81.2%)	53 (88.3%)	
Yes	33 (16.6%)	16 (18.8%)	7 (11.7%)	
Muscle tone				<0.001
Normal	190 (95.5%)	81 (95.3%)	45 (75.0%)	
Hypertonia	9 (4.5%)	4 (4.7%)	15 (25.0%)	
ADL scores (mean ± SD)	48 ± 31	51 ± 31	54 ± 31	0.391
Albumin level (mean ± SD)	36 ± 7	37 ± 6	36 ± 7	0.47
White blood cell count (mean ± SD)	8.6 ± 3.8	9.0 ± 4.5	8.5 ± 6.3	0.83
Barden scores (mean ± SD)	16.3 ± 4.6	16.9 ± 4.5	16.1 ± 5.3	0.462

^1^*n* (%).

^2^Pearson’s Chi-squared test; One-way ANOVA; Fisher’s exact test; BMI, Body Mass Index; ADL, activities of daily living; COPD, chronic obstructive pulmonary disease; DOC, disorders of consciousness; CAD, coronary artery disease.

### 3.2 Risk prediction factors for PI

#### 3.2.1 Preliminary screening of risk predictors

Comparative analysis was conducted to identify risk factors, using variables with significant differences in the training cohort as candidate predictive indicators. The analysis revealed significant associations for stroke subtype (*p* < 0.001), age (*p* < 0.001), and BMI (*p* < 0.001). Variables such as sex (*p* = 0.179), diabetes (*p* = 0.341), hypertension (*p* = 0.99), DOC (*p* = 0.219), CAD (*p* = 0.508), ADL scores (*p* = 0.391), albumin levels (*p* = 0.47), and white blood cell counts (*p* = 0.83) showed no statistically significant differences in the training cohort and were excluded from further modeling. The characteristics of internal and external verification queues is shown in Table 2.

**Table 2:** Univariate logistic regression analysis of risk factors for each group.

Characteristics	Training cohort		*p*-value^2^	Internal validation cohort		*p*-value^3^	External validation cohort		*p*-value^2^
	PI-No, *N* = 136^1^	PI-Yes, *N* = 63^1^		PI-No, *N* = 61^1^	PI-Yes, *N* = 24^1^		PI-No, *N* = 30^1^	PI-Yes, *N* = 30^1^	
Stroke subtype			<0.001			<0.001			0.592
Cerebral hemorrhage	9 (7%)	22 (35%)		2 (3%)	10 (42%)		18 (60%)	20 (67%)	
Cerebral infarction	127 (93%)	41 (65%)		59 (97%)	14 (58%)		12 (40%)	10 (33%)	
Age (median, IQR)	68 (58, 77)	78 (72, 84)	<0.001	74 (66, 78)	80 (73, 85)	0.007	64 (52, 71)	83 (68, 87)	<0.001
Genders			0.179			0.515			0.301
Female	59 (43%)	21 (33%)		25 (41%)	8 (33%)		16 (53%)	12 (40%)	
Male	77 (57%)	42 (67%)		36 (59%)	16 (67%)		14 (47%)	18 (60%)	
BMI (Median, IQR)	23.66 (20.96, 25.39)	21.56 (19.96, 23.47)	<0.001	23.5 (20.8, 25.3)	21.3 (20.0, 23.1)	0.045	24.7 (22.4, 27.0)	18.8 (17.5, 20.8)	<0.001
Diabetes			0.341			0.002			0.317
No	81 (60%)	33 (52%)		40 (66%)	7 (29%)		26 (87%)	23 (77%)	
Yes	55 (40%)	30 (48%)		21 (34%)	17 (71%)		4 (13%)	7 (23%)	
Hypertensive			0.99			0.924			0.592
No	56 (41%)	26 (41%)		21 (34%)	8 (33%)		18 (60%)	20 (67%)	
Yes	80 (59%)	37 (59%)		40 (66%)	16 (67%)		12 (40%)	10 (33%)	
COPD			0.005			0.671			0.112
No	134 (99%)	56 (89%)		56 (92%)	23 (96%)		30 (100%)	26 (87%)	
Yes	2 (1%)	7 (11%)		5 (8%)	1 (4%)		0 (0%)	4 (13%)	
DOC			0.036			<0.001			0.005
No	111 (82%)	43 (68%)		52 (85%)	12 (50%)		30 (100%)	22 (73%)	
Yes	25 (18%)	20 (32%)		9 (15%)	12 (50%)		0 (0%)	8 (27%)	
CAD			<0.001			0.765			0.011
No	123 (90%)	43 (68%)		50 (82%)	19 (79%)		30 (100%)	23 (77%)	
Yes	13 (10%)	20 (32%)		11 (18%)	5 (21%)		0 (0%)	7 (23%)	
Muscle tone			0.468			0.066			0.371
Normal	131 (96%)	59 (94%)		60 (98%)	21 (88%)		21 (70%)	24 (80%)	
Hypertonia	5 (4%)	4 (6%)		1 (2%)	3 (13%)		9 (30%)	6 (20%)	
ADL scores (median, IQR)	65 (40, 80)	20 (0, 35)	<0.001	70 (50, 80)	13 (0, 31)	<0.001	85 (70, 95)	30 (15, 40)	<0.001
Albumin level (median, IQR)	38 (35, 41)	32 (28, 37)	<0.001	38.8 (36.8, 41.3)	32.4 (26.4, 39.4)	0.001	41 (39, 43)	31 (28, 35)	<0.001
White blood cell count (median, IQR)	7.3 (5.8, 8.8)	10.0 (7.5, 13.2)	<0.001	7.0 (5.8, 8.9)	11.4 (7.6, 15.7)	<0.001	7 (5, 8)	7 (6, 10)	0.206
Barden scores (median, IQR)	19.0 (14.0, 21.0)	12.0 (10.0, 14.5)	<0.001	19.0 (17.0, 21.0)	11.5 (10.0, 13.3)	<0.001	21.0 (20.0, 23.0)	11.0 (10.0, 12.0)	<0.001

^1^*n* (%).

^2^Pearson’s Chi-squared test; Wilcoxon rank sum test; Fisher’s exact test.

^3^Fisher’s exact test; Wilcoxon rank sum test; Pearson’s Chi-squared test.

IQR, interquartile range; BMI, Body Mass Index; ADL, activities of daily living; COPD, chronic obstructive pulmonary disease; DOC, disorders of consciousness; CAD, coronary artery disease; PI, pressure injury.

#### 3.2.2 LASSO regression analysis

**Figure 2:**
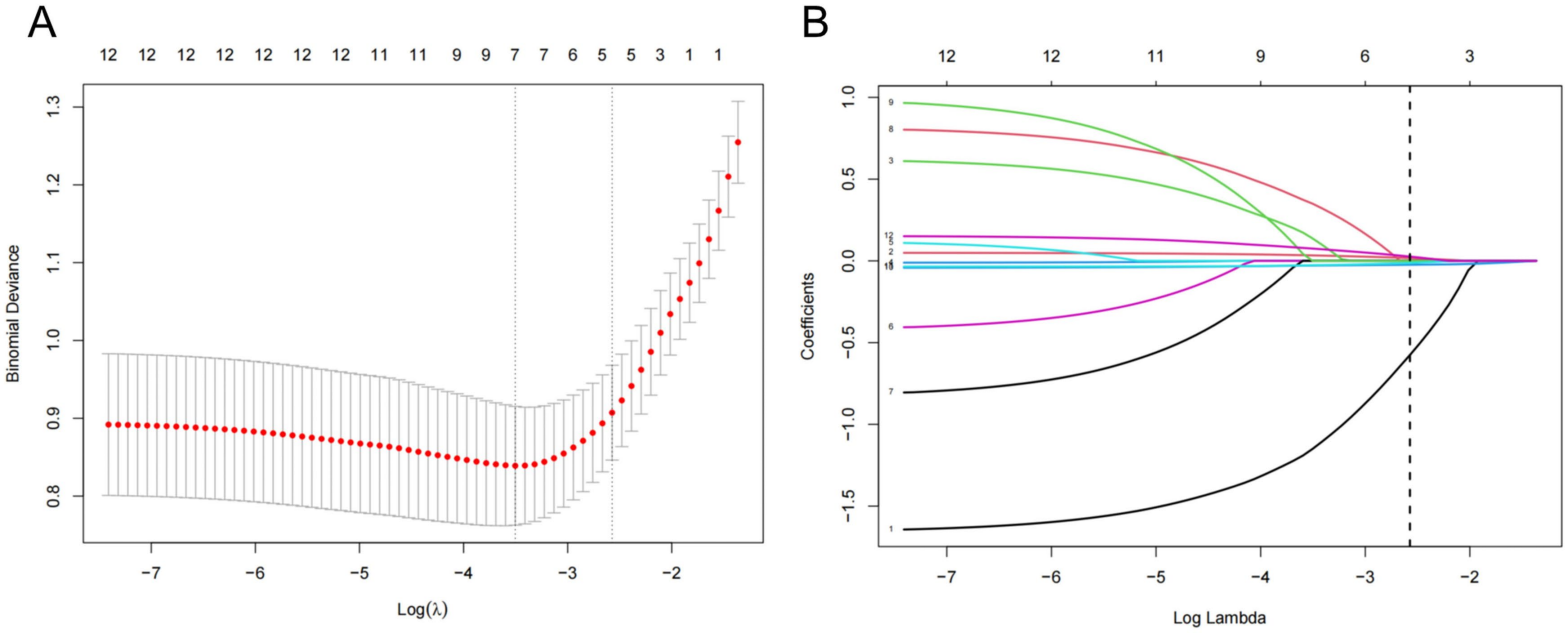
**(A)** Lasso regression cross-validation plot. **(B)** Lasso regression coefficient path plot.

LASSO regression analysis performed in the training cohort reduced these to five potential predictive factors (Figure 2). The most regularized and reasonable model included five variables: stroke type, age, ADL score, albumin level, and white blood cell count, with the cross-validation error within one standard error of the minimum. ROC analyses for these variables yielded the following AUCs: stroke subtype (AUC = 0.642), age (AUC = 0.756), ADL score (AUC = 0.839), albumin level (AUC = 0.754), and white blood cell count (AUC = 0.712).

#### 3.2.3 Multivariate logistic regression analysis

Multivariate logistic regression analysis identified hemorrhagic stroke subtype as a protective factor (OR = 0.17, 95%CI 0.06–0.52, *p* = 0.002) compared to cerebral infarction. Age demonstrated a per-year increase in risk (OR = 1.06, 95%CI 1.02–1.11, *p* = 0.003), while each unit increase in ADL score conferred a 4% risk reduction (OR = 0.96, 95%CI 0.95–0.98, *p* < 0.001). Elevated white blood cell count was positively associated with pressure ulcer development (OR = 1.13 per 1 × 10^9^/L increase, 95%CI 1.01–1.26, *p* = 0.035). No significant association was observed for albumin levels (OR = 0.96, 95%CI 0.90–1.02, *p* = 0.168) (Table 3).

**Table 3:** Results of multivariate logistic regression for training cohort.

Characteristic	*N*	PI	OR^1^	95% CI^1^	*p*-value
Stroke subtype					
Cerebral hemorrhage	31	22	—	—	
Cerebral infarction	168	41	0.17	0.06, 0.52	0.002
Age	199	63	1.06	1.02, 1.11	0.003
ADL scores	199	63	0.96	0.95, 0.98	<0.001
Albumin level	199	63	0.96	0.90, 1.02	0.168
White blood cell count	199	63	1.13	1.01, 1.26	0.035

^1^OR, odds ratio; CI, confidence interval; PI, pressure injury.

ADL, activities of daily living.

### 3.3 Construction of the predictive model

The finalized predictive model incorporated five independent predictors, including stroke subtype, age, ADL assessment, serum albumin concentration, and white blood cell counts, which were subsequently operationalized into a clinically applicable nomogram. This visual analytical tool employs a multidimensional weighting system, translating regression coefficients into standardized scoring metrics through parallel coordinate axes with stratified quantification tiers. The cumulative risk score, derived from summation of variable-specific scores, facilitates the precise quantification of PI incidence probability among hospitalized stroke patients.

**Figure 3:**
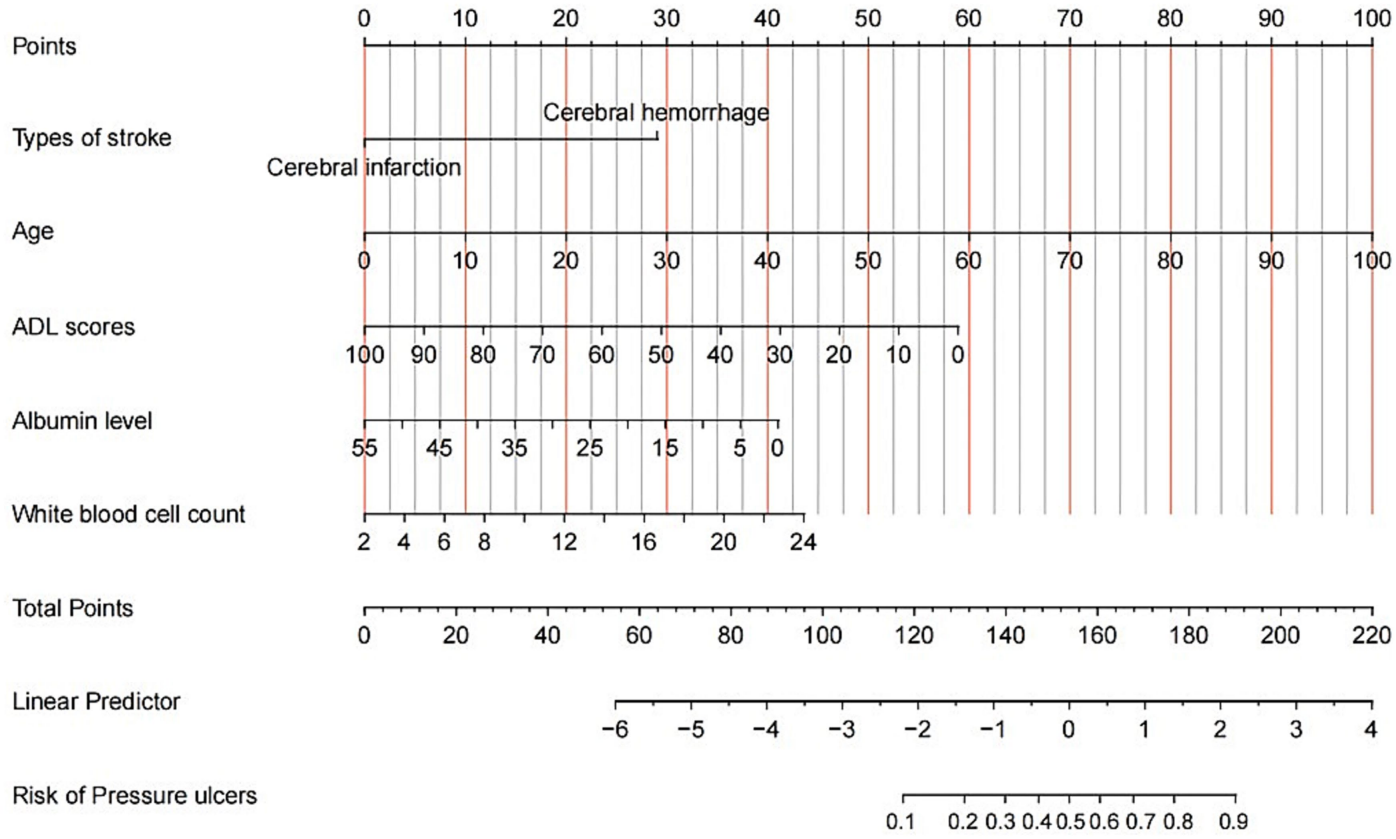
Nomogram prediction model of PI.

Illustratively, a 70-year-old stroke patient presenting with an ADL score of 49, plasma albumin level of 15 g/L, and white blood cell counts of 12 × 10^9/L would be assigned respective scores of 0 (stroke subtype), 70 (age), 30 (ADL), 30 (albumin), and 20 (white blood cell counts), yielding a cumulative risk score of 150 corresponding to a 20% probability of PI development (Figure 3).

### 3.4 Validation of the predictive model

**Figure 4:**
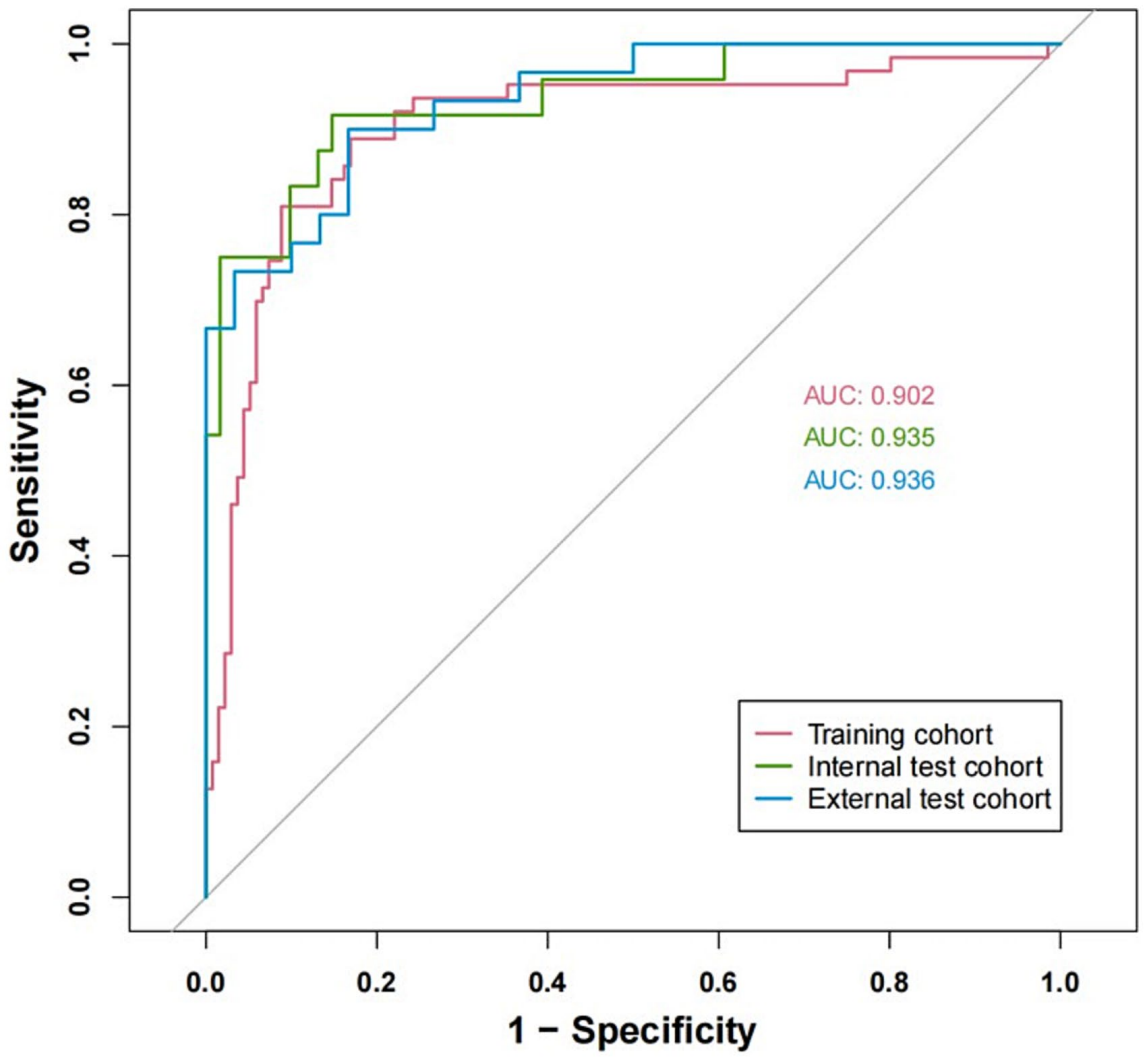
ROC curves of the nomogram in the training cohort, internal validation cohort and external validation cohort.

**Figure 5:**
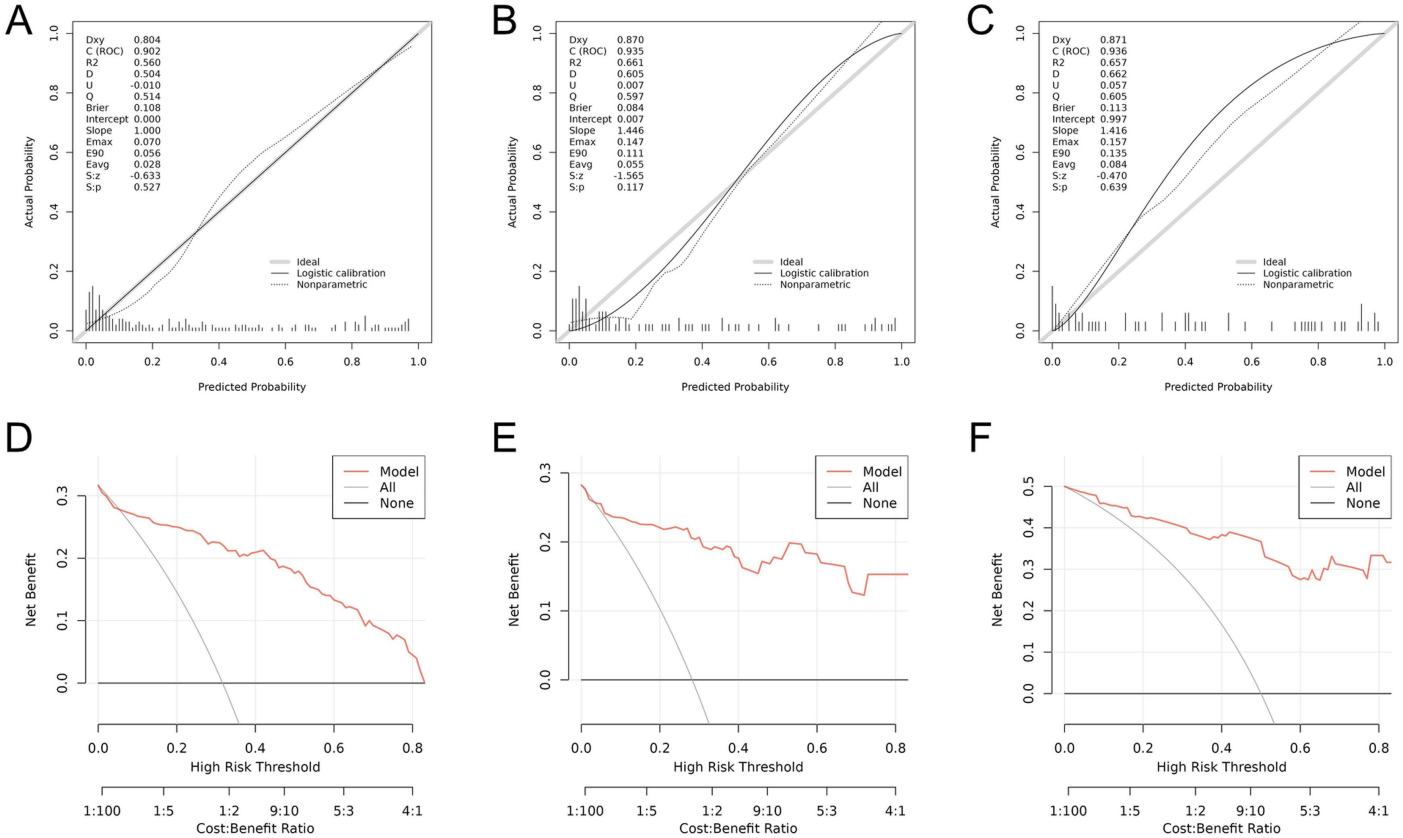
**(A)** Calibration curve of the nomogram prediction model for the training cohort. **(B)** Calibration curve of the nomogram prediction model for the internal test cohort. **(C)** Calibration curve of the nomogram prediction model for the external test cohort. **(D)** Decision curve analysis of the nomogram for the training cohort. **(E)** Decision curve analysis of the nomogram for the internal test cohort. **(F)** Decision curve analysis of the nomogram for the external test cohort.

To ensure the accuracy and reliability of the predictive model, a series of validation methods were employed, including calibration curves and AUC calculations. The AUC for the training cohort was 0.902, for the internal validation cohort was 0.935, and for the external validation cohort was 0.936 (Figure 4). To further assess the model’s validity, a bootstrap method was employed to build calibration curves based on 1,000 resamples of the original data. The results indicated that the nomogram remained effective in the validation cohort, with calibration curves closely aligning with the ideal curve, suggesting that the predicted outcomes were consistent with actual results (see Figures 5A–C).

### 3.5 Decision curve analysis and model performance

Figures 5D–F shows the DCA associated with the nomogram. The high-risk threshold probability indicates that when clinicians encounter significant flaws using the nomogram for diagnostic and decision-making purposes, the model’s predictions may differ significantly. This study demonstrated that the nomogram provides substantial net benefits for clinical applications as indicated by its DCA curve. The predictive classification accuracy of the model was assessed at various risk cutoff points (see Table 4).

**Table 4:** Classification accuracy for prediction at different risk cutoff points for the model in training cohort.

Risk score threshold	Linear predictor cutoff point	Sensitivity (%)	Specificity (%)	PPV (%)	NPV (%)	Accuracy (%)	Precision (%)	Recall (%)	F1
≥0%	-Inf	100	0	31.7		31.7	31.7	100	0.481
≥10%	−2.1972246	95.2	55.1	49.6	96.2	67.8	49.6	95.2	0.652
≥20%	−1.3862944	93.7	72.8	61.5	96.1	79.4	61.5	93.7	0.742
≥30%	−0.8472979	88.9	80.9	68.3	94	83.4	68.3	88.9	0.772
≥40%	−0.4054651	81	89.7	78.5	91	86.9	78.5	81	0.797
≥50%	0	69.8	93.4	83	87	85.9	83	69.8	0.759
≥60%	0.4054651	58.7	94.9	84.1	83.2	83.4	84.1	58.7	0.692
≥70%	0.8472979	47.6	96.3	85.7	79.9	80.9	85.7	47.6	0.612
≥80%	1.3862944	39.7	97.1	86.2	77.6	78.9	86.2	39.7	0.543
≥90%	2.1972246	19	98.5	85.7	72.4	73.4	85.7	19	0.312
≥100%	Inf	0	100		68.3	68.3		0	

PPV, positive predictive value; NPV, negative predictive value.

### 3.6 Comparison of model performance with the Braden scale

**Figure 6:**
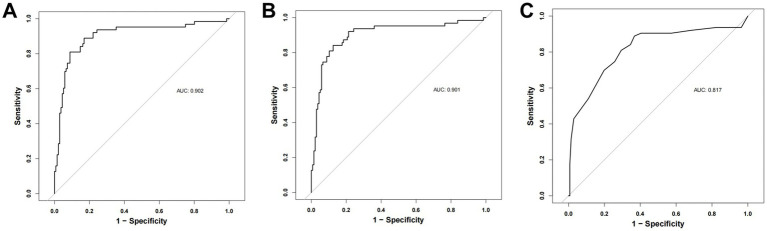
**(A)** ROC curve of the prediction model for the training cohort (Model 1). **(B)** ROC curve after addition of Braden Score (model 2). **(C)** ROC curve of the Braden Score (model 3).

The Braden scale has been extensively utilized for PI risk evaluation. However, its predictive validity may be limited by significant reliance on subjective clinical assessments (5, 15). Both the novel predictive model developed in this study (Model 1) and the composite model integrating Braden Scale parameters (Model 2) demonstrated significantly enhanced predictive accuracy compared to the conventional Braden Scale-based model (Model 3), while exhibiting comparable predictive performance between Models 1 and 2 (Figure 6). Notably, the proposed model achieved equivalent efficacy with fewer predictive variables, demonstrating enhanced operational feasibility and superior clinical applicability.

## 4 Discussion

This study derives its data from multiple centers treating stroke patients in the western region of China, providing a certain level of representativeness. The predictive model developed here can reliably estimate the risk of PI in stroke recovery patients in Asia. Through univariate and LASSO regression analyses, combined with the nomogram approach, it was found that ischemic stroke, increasing age, low albumin levels, low ADL scores, and elevated white blood cell counts are closely associated with the risk of pressure injuries in stroke patients, consistent with previous studies (6, 8).

Ischemic stroke is currently the most common type of stroke. Following ischemic stroke, vascular obstruction leads to insufficient blood supply to brain tissue, resulting in cellular hypoxia and metabolic disturbances (16). This hypoxic state may affect systemic hemodynamics, reducing blood flow to the skin and soft tissues, thereby increasing the risk of PI (17). Additionally, the total white blood cell count is a typical indicator of systemic inflammation, which tends to increase in ischemic strokes. Elevated and activated white blood cells may promote organ ischemia by damaging endothelial cells and increasing oxidative stress. Continuous pressure can also lead to tissue ischemia; after a period of ischemia, restoring blood supply to the affected tissue can cause more severe damage than ischemia alone. Furthermore, reperfusion of ischemic tissue may increase the formation of reactive oxygen species, triggering inflammatory responses that lower physical activity levels, creating a vicious cycle (18, 19).

In agreement with studies by Neziraj et al. (20) and Gurun et al. (21), elderly stroke patients are at a higher risk of developing PI. Older adults are a high-risk group for stroke, often presenting with thinner skin, lower hydration, prolonged bed rest, and multiple comorbidities, which reduce their mechanical resistance to skin damage under pressure or shear forces (22). Aging skin may also exhibit reduced neural supply, impairing the ability to induce vasodilation in response to pressure, leading to early reductions in skin blood flow at very low pressure levels, thus increasing the risk of PI (23, 24).

Patients who have suffered a stroke frequently experience limb mobility impairments and sensory abnormalities, resulting in ADL scores that typically indicate moderate to severe dependence. Many patients have reduced independent mobility due to insufficient muscle strength and find it difficult to move autonomously. During passive transfers and movements, friction and shear forces between the skin and clothing or bed linens can damage the dermal-epidermal junction, lead to desquamation of the stratum corneum, and rupture subcutaneous capillaries, further reducing blood supply to the skin and subcutaneous tissues, thus increasing PI risk (8, 25). Additionally, stroke recovery patients often experience sensory abnormalities or consciousness disturbances, preventing them from perceiving abnormal sensations at pressure points, making them more susceptible to PI (26, 27).

Nutrition is a crucial factor in the prevention and treatment of PI, with albumin serving as a nutritional marker (28, 29). Previous studies have shown that 15 to 40% of older adults are at risk of malnutrition due to declining bodily functions and chronic diseases, making them more susceptible to PI (20). Albumin plays an important role in maintaining osmotic pressure; low albumin levels can decrease osmotic pressure, leading to fluid leakage from blood vessels into tissues, resulting in edema—one of the primary causes of pressure injuries. This is because the tolerance of skin tissue to pressure is reduced; when skin tissue is subjected to pressure or exists in ischemic and hypoxic states, it becomes more vulnerable to damage, thereby increasing the risk of PI (30–32). Current evidence suggests that the Braden Scale may exhibit limitations in predicting rehabilitation outcomes, particularly among patients hospitalized with infectious complications (33, 34). Consequently, early systematic rehabilitation assessment and targeted interventions for geriatric populations presenting with ADL dependency, hypoalbuminemia, or elevated leukocyte counts may contribute to reducing the clinical incidence of PI.

The clinical nomogram is a user-friendly visualization tool based on multifactorial statistical models that quantifies the risk of an event occurring based on various indicators of the study subjects (35). PI are a significant cause of increased morbidity, disability, and mortality among stroke patients, with a high incidence rate. Given the increasing aging population, and the shift in stroke treatment strategies from merely addressing the disease to focusing on functional rehabilitation, it is essential to identify a simple and effective method for predicting the risk of pressure injuries post-stroke. The PI nomogram prediction model is straightforward, integrating biochemical tests with assessment scales. Compared with the machine learning model developed by Staffa and Zurakowski, 2021 (36) (requiring 28 clinical features, AUC = 0.92), our prediction model achieved comparable discriminative performance (AUC = 0.936) using only five routinely measured clinical parameters, significantly enhancing clinical accessibility. When benchmarked against the composite model recommended by the latest meta-analysis (pooled AUC = 0.94) (37), our model demonstrated equivalent predictive accuracy while employing a streamlined variable set. Furthermore, the incorporation of multicenter external validation cohorts confirmed the model’s generalizability, substantially strengthening the tool’s translational potential. The model’s C-index and calibration curves indicate good discrimination and calibration (38), with strong internal and external validation results suggesting good reproducibility and generalizability. Moreover, this model demonstrates higher efficacy than the Braden scale, allowing for easy clinical application either alone or in conjunction with the Braden scale.

The limitations of this study include that the external validation was based on a single dataset. Incorporating samples from different time periods or international datasets as external validation cohorts for temporal and multicenter validation could further enhance the model’s efficacy (39). Additionally, further clinical practice is necessary to validate the predictive effectiveness of this model.

## 5 Conclusion

This study systematically integrated multidimensional clinical indicators including stroke subtype, age, albumin levels, white blood cell count, and ADL scores to construct a validated predictive nomogram for PI risk stratification in stroke rehabilitation patients. Compared to conventional Braden scale assessments, this model demonstrates standalone clinical utility while retaining compatibility with existing evaluation tools, providing a novel approach for predicting PI.

## Data Availability

The datasets presented in this study can be found in online repositories. The names of the repository/repositories and accession number(s) can be found in the article/Supplementary material.

## Footnotes

^1^ www.mstata.com
