# Supplementary figures and images for: Development and validation of a nomogram for pressure injury risk prediction in stroke patients: a retrospective cohort study
Source: Front Neurol. 2025 Sep 9;16:1593707. doi: 10.3389/fneur.2025.1593707 (PMC12456027; doi:10.3389/fneur.2025.1593707)

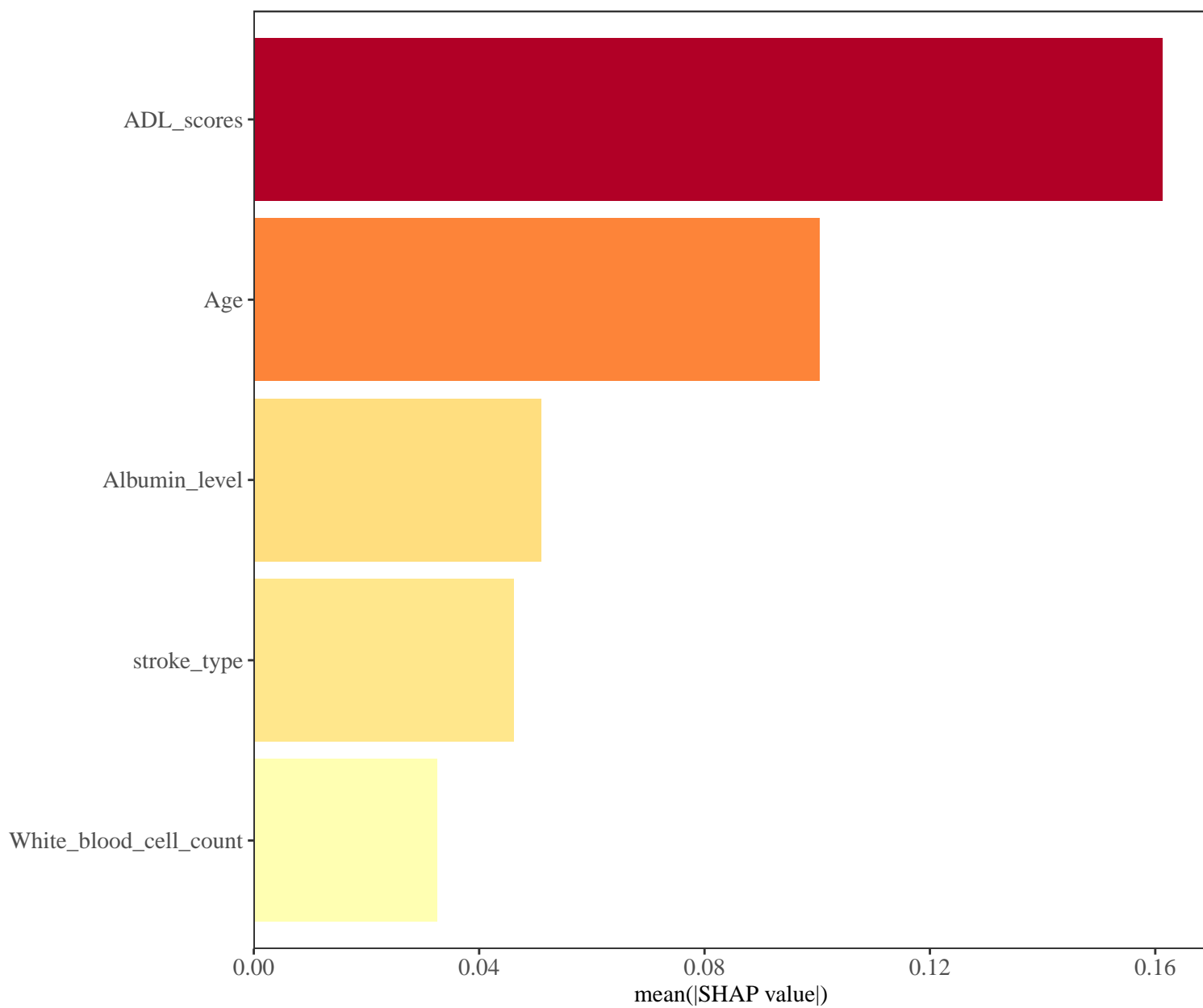

Supplement: Supplementary file 2 [file Data_Sheet_2.pdf]

# Feature Importance

Logistic

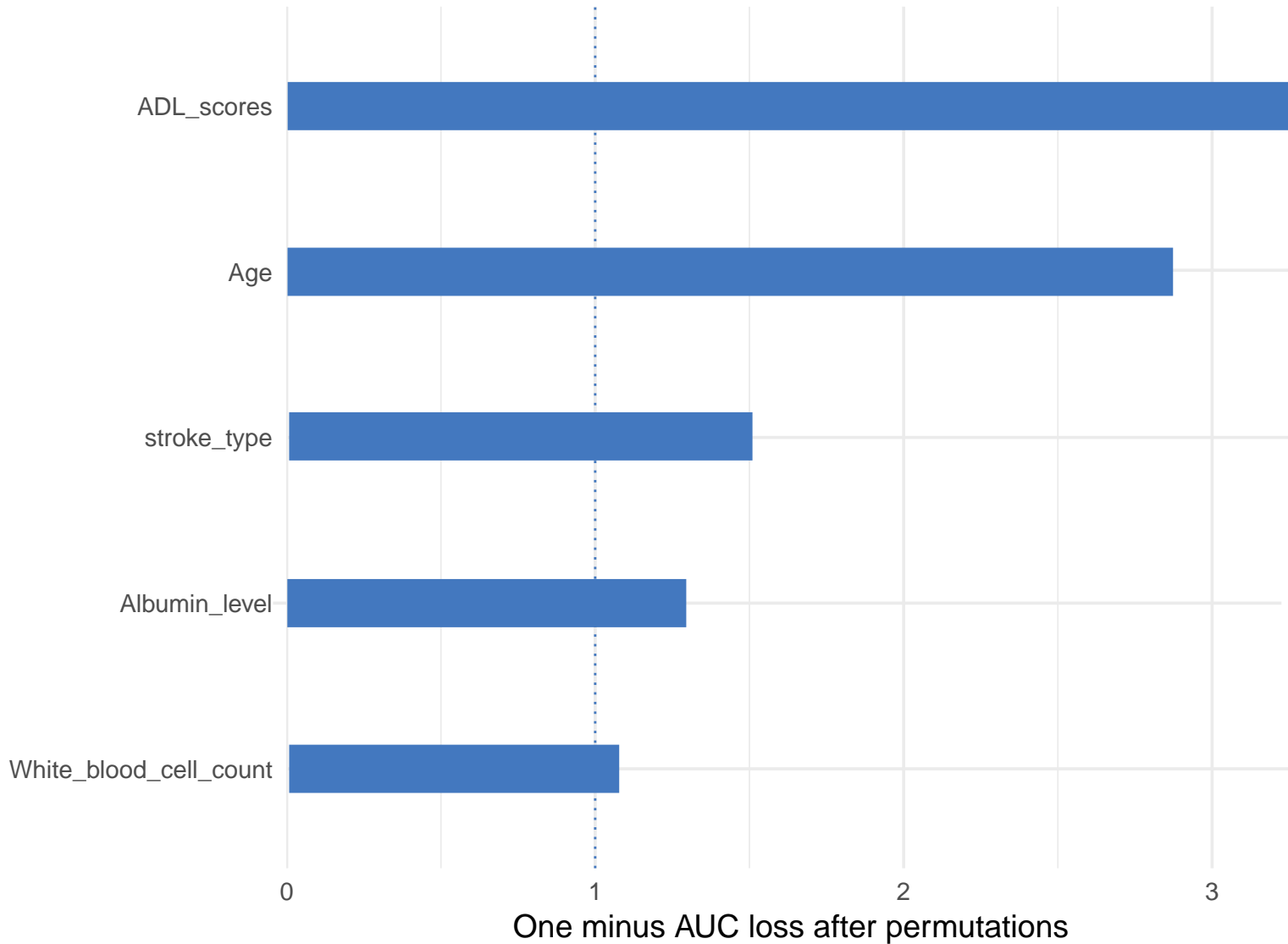

Supplement: Supplementary file 3 [file Data_Sheet_3.pdf]
